# Tweeting for and Against Public Health Policy: Response to the Chicago Department of Public Health's Electronic Cigarette Twitter Campaign

**DOI:** 10.2196/jmir.3622

**Published:** 2014-10-16

**Authors:** Jenine K Harris, Sarah Moreland-Russell, Bechara Choucair, Raed Mansour, Mackenzie Staub, Kendall Simmons

**Affiliations:** ^1^Washington University in St. LouisSt. Louis, MOUnited States; ^2^Chicago Department of Public HealthChicago, ILUnited States

**Keywords:** Twitter, health departments, e-cigarette

## Abstract

**Background:**

In January 2014, the Chicago City Council scheduled a vote on local regulation of electronic cigarettes as tobacco products. One week prior to the vote, the Chicago Department of Public Health (CDPH) released a series of messages about electronic cigarettes (e-cigarettes) through its Twitter account. Shortly after the messages, or tweets, were released, the department’s Twitter account became the target of a “Twitter bomb” by Twitter users sending more than 600 tweets in one week against the proposed regulation.

**Objective:**

The purpose of our study was to examine the messages and tweet patterns in the social media response to the CDPH e-cigarette campaign.

**Methods:**

We collected all tweets mentioning the CDPH in the week between the e-cigarette campaign and the vote on the new local e-cigarette policy. We conducted a content analysis of the tweets, used descriptive statistics to examine characteristics of involved Twitter users, and used network visualization and descriptive statistics to identify Twitter users prominent in the conversation.

**Results:**

Of the 683 tweets mentioning CDPH during the week, 609 (89.2%) were anti-policy. More than half of anti-policy tweets were about use of electronic cigarettes for cessation as a healthier alternative to combustible cigarettes (358/609, 58.8%). Just over one-third of anti-policy tweets asserted that the health department was lying or disseminating propaganda (224/609, 36.8%). Approximately 14% (96/683, 14.1%) of the tweets used an account or included elements consistent with “astroturfing”—a strategy employed to promote a false sense of consensus around an idea. Few Twitter users were from the Chicago area; Twitter users from Chicago were significantly more likely than expected to tweet in support of the policy.

**Conclusions:**

Our findings may assist public health organizations to anticipate, recognize, and respond to coordinated social media campaigns.

## Introduction

The use of electronic cigarettes (e-cigarettes) is growing rapidly in the United States. In a single year, e-cigarette use in the United States doubled for adults [1] and middle and high school students [2]. Adults and youth believe e-cigarettes are less harmful than combustible cigarettes [3,4] and nearly half of Americans believe e-cigarettes should be made available for smoking cessation [4]. Evidence related to e-cigarette effectiveness in promoting smoking cessation is mixed; studies have found increased readiness and confidence to quit after 1 week of e-cigarette use [5] and success in reducing or eliminating smoking in smokers [6,7] while other research found cessation rates were not significantly higher when e-cigarettes were used compared to other cessation aids [8]. While health risks associated with e-cigarette use are not well understood [9,10], a limited number of studies examining the chemical composition of what is inhaled by an e-cigarette user suggests surveillance and additional research is warranted [11-15].

Emerging evidence suggests that e-cigarette use is associated with smoking among adolescents and may encourage smoking initiation [16]. In light of falling smoking rates in the United States, established and emerging tobacco companies are investing in e-cigarettes and other smokeless tobacco to recruit new and retain existing customers [8]. E-cigarette marketing expenditures have increased rapidly in recent years in the United States [17], as has exposure of youth and young adults to e-cigarette advertising [18]. E-cigarettes are marketed on social media [4,19,20], and come in a high-tech format and sweet flavors like cotton candy, which may attract younger consumers [2,4,21,22]. Use of e-cigarettes is increasing in youth and young adults, including in youth and young adults who do not smoke traditional cigarettes [23].

On April 24, 2014, the Food and Drug Administration (FDA) proposed a new rule to extend existing tobacco authority to e-cigarettes and other tobacco products [24]. However, current FDA authority prohibits advertising of e-cigarettes as a therapeutic device for smoking cessation, but does not regulate e-cigarettes as a tobacco product [9]. As of January 3, 2014, three state laws and 108 local laws restricted e-cigarette use in 100% smoke-free venues and nine states restricted use in other venues [25]. On January 15th, 2014, the Chicago City Council voted to regulate e-cigarettes as tobacco products [26]. The regulation defines e-cigarettes as tobacco products and applies Chicago’s tobacco control laws to e-cigarettes (record #SO2013-6160 [27]).

Informing and educating constituents about health is one of 10 essential services provided by local health departments (LHDs) in the United States [28]. Communication with constituents about health and health risks is required of LHDs for accreditation [29]. In past research, 61% of LHDs met standards for informing and educating constituents [30], suggesting a gap between current practices and best practice for communicating with constituents. LHDs have begun to adopt Twitter and other social media for communication, which may help fill the gap [31,32]. Currently, LHDs use social media primarily to broadcast information rather than interact with constituents [33-35].

One week prior to the e-cigarette policy vote, the Chicago Department of Public Health (CDPH) used Twitter to disseminate a series of tweets about e-cigarettes. Shortly after releasing the tweets, the CDPH Twitter feed was “Twitter bombed” by Twitter users tweeting against the proposed policy change. Twitter bombing is a social media strategy designed to fill Twitter feeds with a specific message in order to “establish a false sense of group consensus about a particular idea” and to make the message a trending topic on Twitter [36]. In politics, this strategy has been called “astroturfing”, which is a movement that appears to be grassroots, but is supported by a corporation, industry trade association, political interest group, or public relations firm [37-40]. The tobacco industry has historically used astroturfing by working with third-party allies [37,38] and including citizen-driven groups such as the smokers’ rights movement to unite and oppose tobacco policies [37,40,41].

Astroturfing through social media has been used with some success in elections for and against candidates and issues [36,42,43]. To better understand how social media was used to oppose the Chicago e-cigarette policy, we examined patterns of Twitter use, connections among Twitter users, and content of tweets sent during the week between the campaign and the vote. To our knowledge, this is the first analysis of a Twitter bombing of a public health organization or topic. This information will aid LHDs and other organizations in anticipating, recognizing, and responding to coordinated social media strategies.

## Methods

### Study Context

On January 8, 2014, CDPH conducted a 1-day Twitter campaign about e-cigarettes (Table 1). CDPH Twitter campaigns are typically short, given the many priorities of the department and the varied interests of constituents. CDPH used the hashtag #ECigTruths to facilitate engagement with the tweets. Hashtags (#) are metadata embedded in tweets allowing users to click on the hashtagged word and see all tweets using the same hashtag. Hashtags facilitate group formation around specific topics or events [44] and are positively associated with retweeting [45,46]. The CDPH also directly invited engagement by ending the first e-cigarette tweet with, “Let’s talk about it!”

Throughout January 8^th^, 12 pro-policy and 11 anti-policy tweets were directed to the CDPH Twitter feed by including the CDPH Twitter handle @ChiPublicHealth in the tweet. Including a Twitter handle in a tweet is called “mentioning” [47]. If the mention is *first* in the tweet, the tweet shows up in the Twitter timeline of those who follow both the tweeter and receiver of the mention; if the mention is later in the tweet, it is treated like a regular tweet and shows up for followers of the tweeter. Both types of mentions are visible to the owners of @ChiPublicHealth in the mentions section of the account; if the mention is in a public tweet, the tweet is also publicly available.

At 12:46am January 9^th^, Twitter user @A tweeted the following, “We need to twitter bomb the hell out if [sic] @chiPublicHealth, spreading nothing but lies #kcavo #vaping…[URL].” The tweet was visible to the followers of @A and Twitter users following #kcavo and/or #vaping. The hashtag #kcavo is short for a saying among e-cigarette users, or *vapers*, “keep calm and vape on”. The Twitter profile of @A connects to a professional website that links to pro-vaping advocacy groups, conferences, and an e-cigarette-related business. Over the next 6 days more than 600 tweets were sent opposing the e-cigarette policy and including @ChiPublicHealth.

**Table 1:** E-cigarette tweets sent by the Chicago Department of Public Health (@ChiPublicHealth) on January 8, 2014^a^.

Time	Tweet
10:05am	#ECigs look like, are labeled & contain nicotine like cigarettes. They should be regulated as such. Let's talk about it! #ecigtruths
10:10am	#ECigs come in cotton candy, bubble gum & gummy bear flavors - clearly meant for children [URL] #ecigtruths
10:14am	The “water vapor” from #ECigs contains benzene, nickel, tin, arsenic, formaldehyde & acrolein #ecigtruths [URL]
10:18am	Percentage of middle school and high school students who used e-cigarettes DOUBLED from 2011 to 2012. They must be regulated. #ecigtruths
10:22am	Electronic cigarettes contain a dangerous, addictive drug & should be regulated like other nicotine products #ecigtruths
10:25am	We have a duty to protect our children from ever picking up a nicotine habit #ecigtruths [URL]
10:28am	Youth are particularly susceptible to behavioral advertising [URL] #ecigtruths
10:30am	We do not want to create a new generation of nicotine-addicted residents. It’s time to regulate #ecigtruths [URL]
10:33am	In Chicago, smoking rates are lower than ever. Let’s not reverse decades of life-saving progress #ecigtruths
10:38am	“9 Terribly Disturbing Things About Electronic Cigarettes” [URL] via @HuffPostBiz #ecigtruths
11:34am	Electronic cigs contain a dangerous, addictive drug & should be regulated like other nicotine products #ecigtruths
12:37pm	Safe? #ecigtruths [URL]

^a^See Multimedia Appendix 1 for table with URLs included.

### Data Collection and Management

Using the twitteR package [48] for R version 2.15.2, we collected 684 tweets mentioning @ChiPublicHealth between January 8, 2014 and January 15, 2014. With each tweet, we collected the date and time it was sent, whether it was an original tweet or a native retweet, who sent the tweet, and who composed the tweet. Retweeting is the sharing of tweets originally composed by a different Twitter user. A native retweet is a retweet using an automated function in Twitter; when this function is used, metadata is added to the tweet marking it as a retweet and attaching the handle of the original tweeter. Using NodeXL [49,50], we collected information about each tweeter including number of followers, following, and total tweets sent. Finally, the CDPH tracked social media activity about the policy from non-CDPH sources during the week.

In reviewing the data, we noticed duplicate messages not marked as retweets. We identified these as non-native retweets (retweeted manually rather than using the automated function), coded them as retweets, and attributed each to its original tweeter. To examine how messages spread, retweet network data was visualized and analyzed using Pajek 3.10 [51,52].

### Coding Tweet Content

To examine tweet content, four of the authors read the tweets independently and identified themes. The authors then met to discuss and developed a common set of themes: safety, lies/propaganda, science, flavors, regulation, and issue salience (Table 2). Tweets were assigned as many themes as relevant. We also coded each tweet as in support, opposed to, or unclear on regulating e-cigarettes. Each tweet was coded by two authors. The team met to discuss disagreements and agreed on final classifications. One tweet was not about e-cigarettes and was dropped.

### Data Analysis

Patterns of Twitter use can indicate whether a conversation developed organically or through strategies consistent with astroturfing. Characteristics of accounts used for astroturfing include (1) a high following-to-follower ratio, which indicates that the user follows many others but is not followed [53,54], (2) the use of a combination of letters and numbers in a username [54], and (3) account age, with newer accounts more likely to belong to spammers [55]. We considered following-to-follower ratios more than two standard deviations above the mean (*z*≥1.96) to be high. Tweeters with two or more of these three characteristics we considered likely to be involved in astroturfing. Tweet characteristics can also aid in identifying messages consistent with astroturfing. For example, sending multiple copies of the same tweet that include different shortened versions of the same URL is a strategy used to avoid being identified by Twitter as a spammer [53].

We used descriptive statistics to examine Twitter user characteristics, message content, and Twitter use patterns. We used network analysis and visualization to examine patterns of retweeting. Specifically, we examined the network consisting of Twitter users with retweeting relationships connecting them. A retweet network is directed, with relationship direction representing information flow. For example, if network member B retweeted a message from network member A, the relationship would be depicted A→B since information is traveling from A to B. In a directed network, outdegree centrality measures how many outgoing ties a network member has. In this case, A→B indicates an outdegree of 1 for A, who was retweeted one time by B. Highly central network members aid in the spread or dissemination of information [56]. Tie strength is also influential in dissemination. In a directed network, asymmetric ties are typically weaker relationships, which facilitate dissemination. Mutual ties are stronger and hinder dissemination since both members in the relationship likely already have the same information [57]. To understand the network members and ties facilitating dissemination policy tweets, we examined (1) network members with high outdegree centrality, and (2) the distribution of asymmetric and mutual ties across the network.

## Results

### Summary

Between January 8, 2014 and January 15, 2014, 306 Twitter users mentioning @ChiPublicHealth each sent an average of 0.83 (SD 1.71) original tweets and 1.40 (SD 1.87) retweets. On average, each user had 776 followers (range 0-38,662) and had tweeted 3063 times (range 2-143,118) during the time their account was open. A total of 35 (11.4%) of the 306 user profiles reported their location as the Chicago area and 13 (4.2%) reported Illinois, while 128 (41.7%) were blank or unknown (eg, “a galaxy far far away”), 83 (27.0%) were from another US state, and 48 (15.6%) were from outside the United States. A few accounts had no followers; to compute the following-to-follower ratio, we added one follower to these accounts. The average ratio of following-to-followers was 1.98 (range 0-19). The oldest Twitter account was opened on March 11, 2007; the newest was opened January 14, 2014. Of the 683 tweets, 258 (37.8%) started with @ChiPublicHealth. Finally, #ECigTruths was included in 174 anti-policy and 43 pro-policy tweets.

A total of 105 Twitter user accounts had one or more of the three indicators of astroturfing; 17 Twitter users had significantly higher than average following-to-follower ratios (*z*≥1.96), seven accounts were opened during the time of the Twitter bombing, and 94 accounts had letters and numbers in the handle. In total, 11 Twitter users had two or three characteristics and were therefore considered likely astroturf accounts.

### Tweet Content

Of the 683 tweets, 51 (7.5%) were in support of e-cigarette regulation (pro-policy), while 609 of the 683 tweets (89.2%) were opposed (anti-policy), and 23 of the 683 tweets (3.4%) were coded as unable to tell. Tweet sentiment was significantly associated with location (χ^2^_4_=95.9; *P*<.001), with standardized residuals indicating Chicago residents were significantly more likely than expected to send a pro-policy tweet. Specifically, 37 Twitter users sent at least one pro-policy tweet; 21 (56.8%) of these were located in Chicago, while 4 were elsewhere in Illinois, 10 were in unknown locations, and two were in other states.

Pro-policy tweets were most likely to focus on regulation (n=44) and science (n=19), while anti-policy tweets were most likely to focus on safety (n=358) and lies/propaganda (n=224). Tweets classified as lies/propaganda included several types of arguments diverting attention from the original e-cigarette messages rather than addressing message substance. For example, a few lies/propaganda tweets resorted to name calling (eg, “@ChiPublicHealth are a bunch of IGNORANT LIARS”) while many focused on discrediting or attacking CDPH rather than addressing the substance of the e-cigarette messages (eg, “@ChiPublicHealth You can’t be that stupid. Typical Chicago corruption. Who owns you?”).

Retweeting was highest in the safety category for all tweeters comprising the 86% of the pro-policy tweeting on safety (12 of 14 tweets), and 76% of the anti-policy safety tweeting (271 of 358 tweets). Science was the second most retweeted topic for all tweeters, with retweeting comprising 84% of pro-policy (16 of 19 tweets) and 71.6% of anti-policy (156 of 218 tweets) tweeting. Table 2 shows the number of tweets and retweets by sentiment and category.

**Table 2:** Themes in e-cigarette tweets mentioning the Chicago Department of Public Health in January, 2014^a^.

Theme	Sentiment	Definition	Example tweet	Alln=683	Tweetsn=255	Retweetsn=428
				n (%)		
**Safety**						
	Pro-policy	e-cigarettes are harmful, foster nicotine addiction, promote smoking	RT @ChiPublicHealth: Electronic cigs contain a dangerous, addictive drug & should be regulated like other nicotine products #ecigtruths htt…	14 (2.0%)	2 (0.8%)	12 (2.8%)
	Anti-policy	e-cigarettes are safer than alternative, promote cessation	@ChiPublicHealth it’s not about being safe, it’s about being SAFER than the alternative #EcigsSaveLives it’s about HARM REDUCTION #Casaa	358 (52.4%)	87 (34.1%)	271 (63.3%)
**Lies/propaganda**						
	Pro-policy	Propaganda/ lie spread by e-cigarette industry or supporter	N/A	0 (0%)	0 (0%)	0 (0%)
	Anti-policy	Propaganda/ lie spread by health department or other government	@ChiPublicHealth Baseless nonfactual propaganda anyone? So much for public health. #GetAClue #ecigtruths #LiesToldOnTwitter	224 (32.8%)	108 (42.4%)	116 (27.1%)
**Science**						
	Pro-policy	Studies find some ingredients are carcinogenic, increased use by kids; need more research	@AmerAcadPeds @ChiPublicHealth Time for local & FDA action to protect youth from e-cigs & toxins in both vapor and smoke #SGR50 #putkids1st	19 (2.8%)	3 (1.2%)	16 (3.7%)
	Anti-policy	Science shows e-cigarettes contain only nicotine and water, no dangerous secondhand vapor	@ChiPublicHealth “Vaping: it’s not smoking [URL]” No smoke. No carcinogens. No shame. #EcigsSaveLives #Casaa #IMPROOF	218 (31.9%)	62 (24.3%)	156 (36.4%)
**Flavor**						
	Pro-policy	Sweet flavors are for kids	RT @ChiPublicHealth: “9 Terribly Disturbing Things About Electronic Cigarettes” [URL] via @HuffPostBiz #ecigtruths	2 (0.3%)	0 (0%)	2 (0.5%)
	Anti-policy	Adults like flavors too	@choucair @ChiPublicHealth no one advocates children smoking. that said, my favorite flavor is of strawberries and watermelon. i cant enjoy?	25 (3.7%)	20 (7.8%)	5 (1.2%)
**Regulation**						
	Pro-policy	Ingredients, look, and use are like cigarettes, should be regulated like cigarettes	RT @IllinoisAFP: @ChiPublicHealth If it looks like a cigarette & contains nicotine like a cigarette, it should be regulated like a cigarett…	44 (6.4%)	10 (3.9%)	34 (7.9%)
	Anti-policy	Regulation is a slippery slope, do not need nanny state	@IllinoisAFP @ChiPublicHealth it looks like a gun, has a trigger like a gun. Let’s ban nerf and water guns!	169 (24.7%)	61 (23.9%)	108 (25.2%)
**Issue salience**						
	Pro-policy	E-cigarettes are an important threat to public health	N/A	0 (0%)	0 (0%)	0 (0%)
	Anti-policy	Health department should focus on more serious health threats	@ChicagosMayor @ChiPublicHealth Y not focus on more free hlth care N staff at the safe passage. Protect kids by actually protecting them.	16 (2.3%)	8 (3.1%)	8 (1.9%)

^a^See Multimedia Appendix 2 for table with URLs included.

### Retweet Network

**Figure 1:**
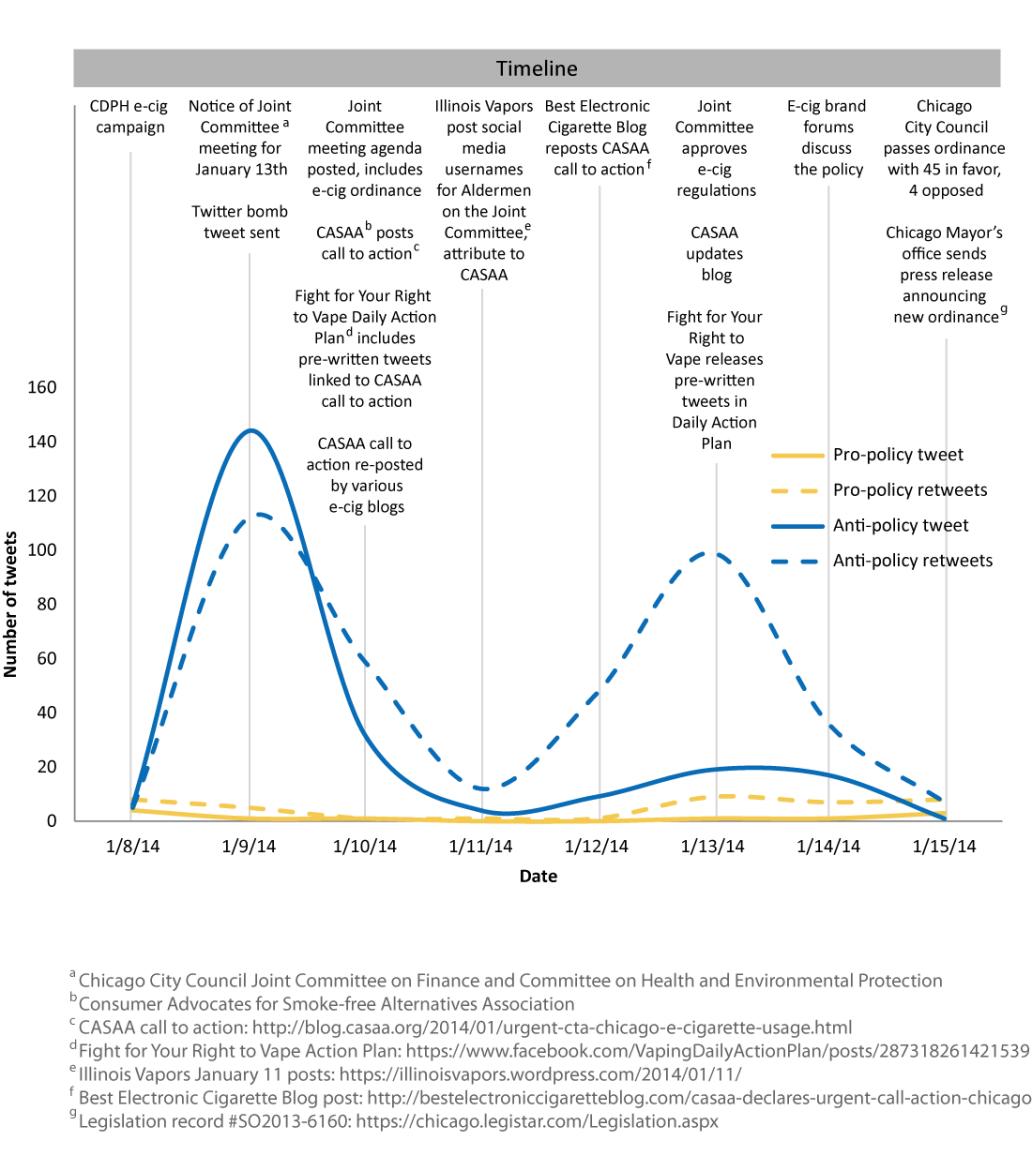
Timeline showing social media activities and distribution of tweets and retweets directed to @ChiPublicHealth in support of and opposing regulation of e-cigarettes in Chicago.

Of the 683 tweets, 62.7% (n=429) were retweets. The majority of the anti-policy tweets were sent January 9^th^, while anti-policy retweets were frequent on the 9^th^ and again on January 13^th^, two days before the city council vote. Pro-policy tweets and retweets were infrequent throughout the week. The distribution of anti- and pro-policy tweets and retweets is shown in Figure 1, which also includes non-CDPH social media events related to the Twitter bombing. For example, on January 10^th^, the Chicago City Council Joint Committee on Finance and Committee on Health and Environmental Protection posted the agenda for their January 13^th^ meeting; the agenda included the e-cigarette policy. Soon after the agenda was posted, Consumer Advocates for Smoke-free Alternatives Association (CASAA), posted an “URGENT CALL TO ACTION” listing strategies to oppose the e-cigarette policy. Figure 1 shows CASAA involvement in a number of the anti-policy social media activities throughout the week.

The retweet network consisted of 259 Twitter users connected to at least one other network member by a retweet link and 47 isolated Twitter users not involved in retweeting. There were 361 asymmetric ties and five mutual ties in the network. Four of the five mutual ties were between the Twitter user labeled “@A” and other network members, and the fifth mutual tie was between @B and @C.

**Figure 2:**
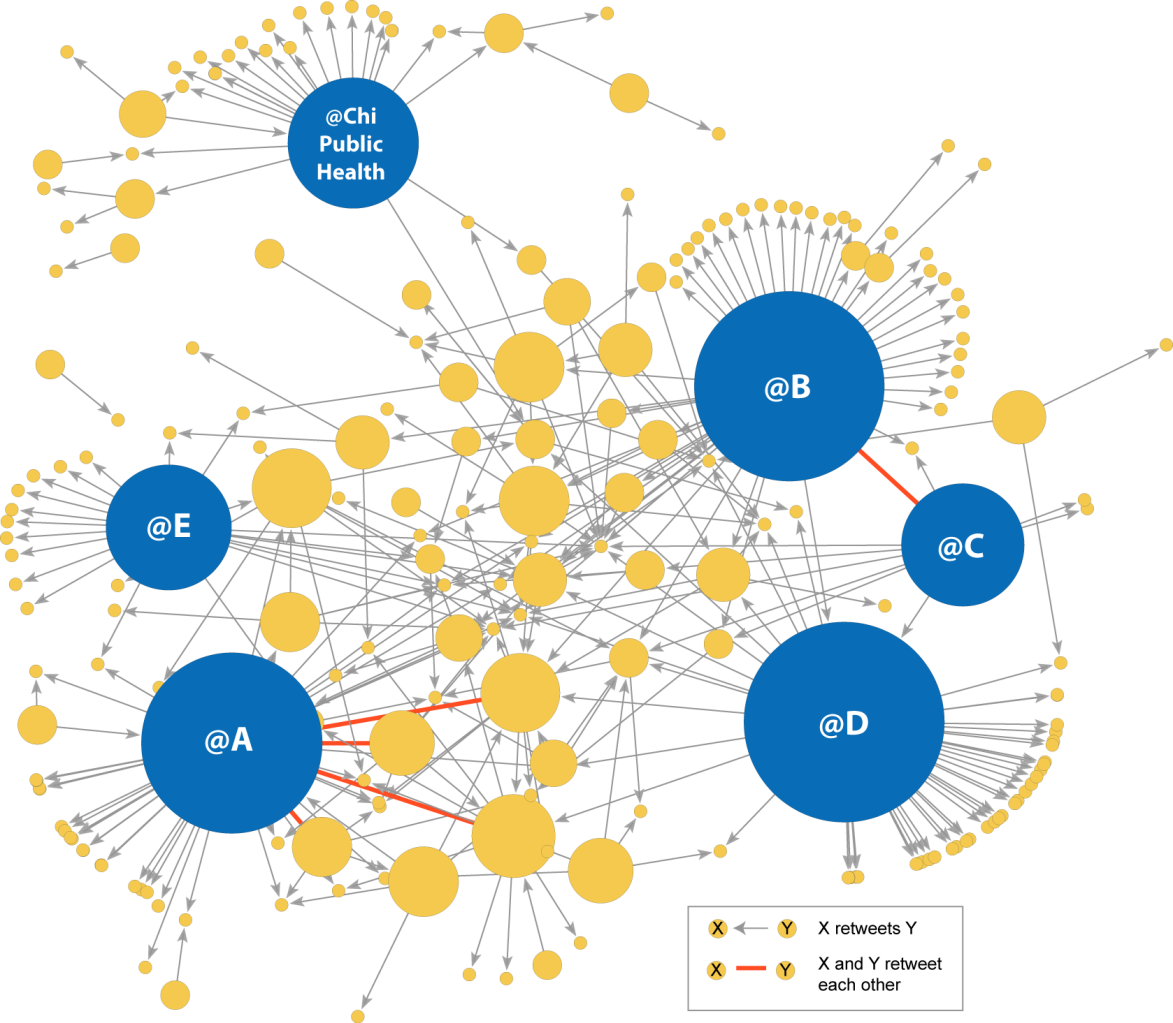
Retweet network with nodes sized by outdegree, or number of times the Twitter user was retweeted during the week. The 6 most highly retweeted network members shown in blue.

Figure 2 shows the network with nodes sizes representing outdegree centrality; the larger nodes were retweeted more often. Six network members had extremely high outdegree centrality. Specifically, Twitter users represented by nodes @A-@E and @ChiPublicHealth were retweeted 22-59 times; no other network member was retweeted more than 10 times. Four of the central Twitter users were affiliated with formal e-cigarette businesses or advocacy groups.

An examination of the six highly retweeted network members found a single original tweet from @B was retweeted 53 times (RT-1), each time using a different shortened URL, with all URLs directed to the same place. The same strategy was used by @C, whose original tweet (RT-2) was retweeted 24 times using different shortened URLs directed to the same place. Changing the shortened URL in a repeated tweet is a known strategy used to avoid spam detection on Twitter [53]. A total of 77 retweets of tweets from @B and @C were sent using the same strategy; @B and @C were also connected by one of very few strong mutual ties in the network.

The #publichealth gain is going to be very, very large-biggest likely this century [URL] @ChiPublicHealth #EcigTruths

RT-1

This tweet (RT-1) linked to a newspaper article outlining the debate over e-cigarettes focusing on safety concerns, harm reduction arguments, discussion of laws banning their use, and their increasing popularity (sent by @B; retweeted 53 times).

@ChiPublicHealth #Please review all of the science before condemning smokers to #QuitOrDie [URL] #ECigTruths

RT-2

This tweet (RT-2) linked to a peer-reviewed journal article about the chemistry of contaminants in e-cigarettes [11] (sent by @C; retweeted 24 times). RT-2 included a link to a peer-reviewed journal article recommended in the CASAA January 10th call to action; the work in the paper was funded by CASAA [11] (see Acknowledgements section of that paper). Overall, 59 retweets, all in the science topic category, linked to this article. The Twitter profile of @B included a link to CASAA, while the profile for @C listed e-cigarette advocacy, but did not include a group affiliation. The Twitter profiles of @B and @C indicated they were not from Chicago. The Twitter profile of the most frequently retweeted network member, @D, lists an e-cigarette retailer as employer; @D sent four original tweets (RT-3 through RT-6) garnering 69 retweets from 59 unique users.

#ecigs Save lives. NO SMOKE WITHOUT FIRE @ChiPublicHealth

RT-3

RT-3 linked to a photo showing a combustible cigarette on the left and an e-cigarette on the right with a list in the middle comparing the chemicals in smoke and vapor (retweeted 6 times).

Bought this lollipop myself at pharmacy at pediatrician office. #ecigs save lives. Lollipops DO NOT @ChiPublicHealth

RT-4

RT-4 linked to a photo displaying a nicotine lollipop with a caption comparing the harm reduction effectiveness and reception of nicotine lollipops to e-cigarettes (retweeted 43 times).

[URL] @ChiPublicHealth #ecigs save lives

RT-5

RT-5 linked to a photo showing a cartoon character saying, “Are you saying that you lie to people about ecigs so that they will not use them even though they work and are safer?” (retweeted 20 times).

[URL] @ChiPublicHealth

RT-6

RT-6 linked to a photo of a crumpled combustible cigarette with a caption stating e-cigarettes are the best harm reduction alternative to smoking but big corporations are paying the FDA to over-regulate them (not retweeted).

A total of 11 different tweets from @A were retweeted between one and 12 times. The original Twitter bomb tweet, sent by @A, was retweeted nine times. Another central user, @E, is a Twitter account representing an online community of “e-cigarette users and campaigners”. @E sent a single tweet that was retweeted 23 times by 23 different Twitter users. The URL in the tweet was to the published study [11] recommended in the January 10^th^ CASAA call to action; the URL was truncated in all 23 retweets.

RT @E: @ChiPublicHealth New paper shows no danger fm "second hand vape": Great news, you can stop worrying now. [truncated URL]…

RT-7

The original link pointed to the *BMC Public Health* journal (see RT-2).

Finally, @ChiPublicHealth was also central in the retweet network with 29 pro-policy, and three anti-policy, retweets.

## Discussion

### Principal Findings

In the week leading up to a vote on local e-cigarette policy by the Chicago City Council, 683 tweets and retweets about e-cigarettes including a mention of @ChiPublicHealth were sent by 307 Twitter users. The majority of the tweets and retweets were against the policy (89.2%, 609/683), and a majority of Twitter users reporting a location in their profile were from states outside Illinois (n=83) or outside the United States (n=48). Twitter users located in Chicago (n=35) were significantly more likely than expected to tweet in favor of the policy.

The CDPH and five Twitter users tweeting against the policy were central in the network of retweeting during this week. Retweet patterns were consistent with past research, which has identified argument quality and source as factors associated with retweeting [58]. In this case, four of the five central retweet network members were affiliated with e-cigarette businesses or advocacy groups, which may have credibility among supporters of e-cigarette use. Safety and science tweets were retweeted more than lies/propaganda, perhaps due to the lower quality arguments (eg, name calling) in the lies/propaganda tweets.

The majority of tweets appeared to be from legitimate Twitter users who oppose the regulation of e-cigarettes, and at least one advocacy group (CASAA) aiming to organize policy opposition messaging. However, our results suggested that 96 of the 683 tweets (14.1%) sent by 73 of the 307 Twitter users (23.8%) were using an account or tweet strategy consistent with astroturfing. The structure of the retweeting network was also consistent with findings from a study of astroturfing where a small number of accounts were responsible for a large proportion of retweets contributing to trending topics [55].

Recent studies of grassroots efforts found email and phone contact with legislators to have a substantial influence on legislative voting behavior [59,60]. Although there are no studies of social media influence on legislative decision making that we know of, a 2011 study found astroturfing has been successful in influencing public opinion [61]. In addition, a 2011 survey of legislative staffers indicated that Twitter is effective in reaching legislators [62], and a 2014 study found that constituents lobby via Twitter [63]. During the Twitter bombing of the CDPH, Chicago City Council members were also a target of the Twitter bombing; City Council members Twitter handles were posted January 11^th^ on the Illinois Vapors blog (see Figure 1). Local health departments and local policy-makers in New York City and Los Angeles have also been targeted via social media by e-cigarette advocates. In Chicago, the Twitter anti-policy campaign did not appear to influence the vote, which was 45-4 in favor of regulating e-cigarettes. However, given the widespread use of Twitter by policy-makers and the potential of grassroots efforts to influence legislative decision making, it is increasingly important to understand and address social media policy advocacy strategies and how they may influence development and support of public health policy.

Advocacy efforts that appear to include astroturfing should be fairly easy to detect. To identify accounts associated with astroturfing, review tweets for (1) different shortened URLs pointing to the same place, and (2) Twitter users who have more than one of the characteristics associated with astroturfing (ie, new account, letters and numbers in the username, and few followers). Removing central nodes from a network is a strategy that has been used to disrupt crime and disease transmission networks [64,65], however, to our knowledge it has not yet been studied as a way to address astroturfing. There are two ways to report Twitter users who seem to be involved in astroturfing or spamming, which will prompt Twitter to review the account and possibly remove it: (1) click “report as spam” link appearing with the offending tweet, or (2) post a tweet that includes “@spam @username” where the username of the spammer is included.

The emergence of social media efforts by the tobacco industry [21] and other industries with products promoting risky health behaviors [66] suggest that new social media strategies are needed to combat novel marketing efforts and to increase the presence of public health on social media platforms. In the case of the Chicago local e-cigarette policy, the number of tweets against the policy was more than 10 times higher than the number of pro-policy tweets despite high policy support from local tweeters. As in offline tobacco use prevention and control, online tobacco use prevention and control may wish to take note of the strategies employed by pro-tobacco interests and adapt them to develop effective counter-marketing. For example, while astroturfing is not an appropriate strategy for public health, tactics employed in astroturfing such as coordinated, widespread, and constant use of specific hashtags or messages (like those supplied by CASAA) to elevate a public health topic on Twitter might work to engage the public around a public health topic. Developing messages that include high quality arguments and originate with reputable sources may aid in increasing message spread [58].

### Conclusions

New media marketing strategies present both challenges and opportunities for public health [67]. There is evidence that misinformation spreads easily on the Internet, especially in social media [68,69]. In addition, although evidence of behavior change resulting from social media use is limited and mixed in public health [70,71], emerging successes with public health interventions that engage participants [71,72], and evidence of an association between Facebook content and smoking and alcohol use in adolescents [73], reinforce the importance of understanding social media engagement with substance promoting and health promotion messages. Efforts to develop the evidence base for social media use in public health are underway through funding opportunities such as the National Institutes of Health 2014 Request for Applications, entitled *Using social media to understand and address substance use and addiction* (RFA-CA-14-008 and RFA-CA-14-009). As social media use continues to grow, additional research is needed to better understand how to develop and implement effective pro-health social media campaigns that engage the public to improve health.

## Multimedia Appendix 1

E-cigarette tweets sent by the Chicago Department of Public Health (@ChiPublicHealth) on January 8, 2014.

## Multimedia Appendix 2

Themes in e-cigarette tweets mentioning the Chicago Department of Public Health in January, 2014.

## Abbreviations

CASAA: Consumer Advocates for Smoke-free Alternatives Association
CDPH: Chicago Department of Public Health
FDA: Food and Drug Administration
LHD: local health departments
