# Supplementary material for: Tweeting for and Against Public Health Policy: Response to the Chicago Department of Public Health's Electronic Cigarette Twitter Campaign
Source: J Med Internet Res. 2014 Oct 16;16(10):e238. doi: 10.2196/jmir.3622 (PMC4210950; doi:10.2196/jmir.3622)
Supplement: Supplementary file 1 [file jmir_v16i10e238_app1.pdf]

Multimedia Appendix 1

E-cigarette tweets sent by the Chicago Department of Public Health (@ChiPublicHealth) on January 8, 2014.

Time	Tweet
10:05am	#ECigs look like, are labeled & contain nicotine like cigarettes. They should be regulated as such. Let's talk about it! #ecigtruths
10:10am	#ECigs come in cotton candy, bubble gum & gummy bear flavors - clearly meant for children http://ow.ly/so0O0 #ecigtruths
10:14am	The "water vapor" from #ECigs contains benzene, nickel, tin, arsenic, formaldehyde & acrolein #ecigtruths http://ow.ly/so1gM
10:18am	Percentage of middle school and high school students who used e-cigarettes DOUBLED from 2011 to 2012. They must be regulated. #ecigtruths
10:22am	Electronic cigarettes contain a dangerous, addictive drug & should be regulated like other nicotine products #ecigtruths
10:25am	We have a duty to protect our children from ever picking up a nicotine habit #ecigtruths http://ow.ly/so3Ae
10:28am	Youth are particularly susceptible to behavioral advertising http://ow.ly/so4cn #ecigtruths
10:30am	We do not want to create a new generation of nicotine-addicted residents. It's time to regulate #ecigtruths http://ow.ly/so4rC
10:33am	In Chicago, smoking rates are lower than ever. Let's not reverse decades of life-saving progress #ecigtruths
10:38am	"9 Terribly Disturbing Things About Electronic Cigarettes" http://huff.to/1dDFg9C via @HuffPostBiz #ecigtruths
11:34am	Electronic cigs contain a dangerous, addictive drug & should be regulated like other nicotine products #ecigtruths
12:37pm	Safe? #ecigtruths pic.twitter.com/f2m4Gps7BD
