# Supplementary material for: Tweeting for and Against Public Health Policy: Response to the Chicago Department of Public Health's Electronic Cigarette Twitter Campaign
Source: J Med Internet Res. 2014 Oct 16;16(10):e238. doi: 10.2196/jmir.3622 (PMC4210950; doi:10.2196/jmir.3622)
Supplement: Supplementary file 2 [file jmir_v16i10e238_app2.pdf]

Multimedia Appendix 2

Themes in e-cigarette tweets mentioning the Chicago Department of Public Health in January, 2014.

Theme	Sentiment	Definition	Example tweet	All n=683	Tweets n=255 n (%)	Retweets n=428
Safety						
	Pro-policy	e-cigarettes are harmful, foster nicotine addiction, promote smoking	RT @ChiPublicHealth: Electronic cigs contain a dangerous, addictive drug & should be regulated like other nicotine products #ecigtruths htt…	14 (2.0%)	2 (0.8%)	12 (2.8%)
	Anti-policy	e-cigarettes are safer than alternative, promote cessation	@ChiPublicHealth it's not about being safe, it's about being SAFER than the alternative #EcigsSaveLives it's about HARM REDUCTION #Casaa	358 (52.4%)	87 (34.1%)	271 (63.3%)
Lies/propaganda						
	Pro-policy	Propaganda/ lie spread by e-cigarette industry or supporter	N/A	0 (0%)	0 (0%)	0 (0%)
	Anti-policy	Propaganda/ lie spread by health department or other government	@ChiPublicHealth Baseless nonfactual propaganda anyone? So much for public health. #GetAClue #ecigtruths #LiesToldOnTwitter	224 (32.8%)	108 (42.4%)	116 (27.1%)
Science						
	Pro-policy	Studies find some ingredients are carcinogenic, increased use by kids; need more research	@AmerAcadPeds @ChiPublicHealth Time for local & FDA action to protect youth from e-cigs & toxins in both vapor and smoke #SGR50 #putkids1st	19 (2.8%)	3 (1.2%)	16 (3.7%)
	Anti-policy	Science shows e-cigarettes	@ChiPublicHealth "Vaping: it's not smoking	218 (31.9%	62 (24.3%	156 (36.4%)

		contain only nicotine and water, no dangerous secondhand vapor	http://t.co/Kkinhzb9JU" No smoke. No carcinogens. No shame. #EcigsSaveLives #Casaa #IMPROOF	)	)	
Flavor						
	Pro-policy	Sweet flavors are for kids	RT @ChiPublicHealth: "9 Terribly Disturbing Things About Electronic Cigarettes" http://t.co/IhMWnol1J7 via @HuffPostBiz #ecigtruths	2 (0.3%)	0 (0%)	2 (0.5%)
	Anti-policy	Adults like flavors too	@choucair @ChiPublicHealth no one advocates children smoking. that said, my favorite flavor is of strawberries and watermelon. i cant enjoy?	25 (3.7%)	20 (7.8%)	5 (1.2%)
Regulation						
	Pro-policy	Ingredients, look, and use are like cigarettes, should be regulated like cigarettes	RT @IllinoisAFP: @ChiPublicHealth If it looks like a cigarette & contains nicotine like a cigarette, it should be regulated like a cigarett…	44 (6.4%)	10 (3.9%)	34 (7.9%)
	Anti-policy	Regulation is a slippery slope, do not need nanny state	@IllinoisAFP @ChiPublicHealth it looks like a gun, has a trigger like a gun. Let's ban nerf and water guns!	169 (24.7%)	61 (23.9%)	108 (25.2%)
Issue salience						
	Pro-policy	E-cigarettes are an important threat to public health	N/A	0 (0%)	0 (0%)	0 (0%)
	Anti-policy	Health department should focus on more serious health threats	@ChicagosMayor @ChiPublicHealth Y not focus on more free hlth care N staff at the safe passage. Protect kids by	16 (2.3%)	8 (3.1%)	8 (1.9%)

actually protecting them.
